# Iritis—Atropia—Paracentesis—Topical Use of Calomel—Aneurism of the Orbit—Sympathetic Ophthalmitis

**Published:** 1872-10

**Authors:** E. L. Holmes

**Affiliations:** Chicago


ARTICLE V.—*Iritis—Atropia—Paracentesis—Topical Use of Calomel—Aneurism of the Orbit—Sympathetic Ophthalmitis.* Read at the meeting of the State Medical Society, held at Rockford, May, 1872, by E. L. HOLMES, M.D., of Chicago.

There are a few topics connected with the diagnosis and treatment of certain common diseases which are of great importance and are yet neglected by many practitioners.

### IRITIS.

It seems to me our best works scarcely dwell with sufficient emphasis on the difficulties which may embarrass the student in the diagnosis of this disease.

A typical case of iritis, even in its early stages, offers little difficulty. The symptoms upon which a diagnosis is based, are irregularity, contraction, and immobility of the pupil—discoloration of the iris, with a loss of the brilliancy of its surface, turbid condition of the aqueous humor, pink zone around the cornea, photophobia in and about the eye, pain, and diminution of sight from turbidity of the aqueous humor, or exudation upon the crystalline lens.

Not unfrequently these symptoms are masked. From the very onset there may be not only considerable œdema of the conjunctiva of the globe, but also of the whole lid, with no marked change in the appearance of the iris or pupil. Such an attack could be easily mistaken for simple conjunctivitis. The terrible pain, so often experienced in iritis, is sometimes absent. The pupil is not always, at the commencement, irregular. In some forms of the disease, the inflammation is confined almost entirely to the posterior layers of the iris, producing no exudation upon the pupillary edge, and scarcely any redness or irregularity.

Many of the symptoms of iritis are present in some forms of keratitis. We have redness, photophobia, contracted and immovable pupil, dimness of vision, and apparent turbidity of the aqueous humor, with apparent want of brilliancy of the surface of the iris from a cloudy condition of the cornea.

I allude to this subject, from the fact that vision of very many eyes is partially, or even totally, lost, because an improper diagnosis is made, and, in consequence, injurious treatment instituted. It is seldom that a mistake would be made, if the practitioner would take the trouble, in cases where doubt is possible, to instil a drop or two of solution of *atropiæ sulph.* (gr. iv to the ounce) into the eye. If iritis is present there is scarcely a possibility that the pupil will not dilate irregularly, which is, perhaps, the most important symptom in diagnosticating iritis.

I do not wish to dwell on the treatment of iritis, but will simply add that the free local use of atropiæ sulph. is of chief importance. The patient should be kept at rest, free from exposure to light. Pain may be relieved by subcutaneous injections of morphine. There is reason to believe that mild but continued doses of calomel serve, in a great degree, to shorten the course of the disease, although it is far inferior in value to atropine.

### ATROPIÆ SULPH.

I wish to ask the attention of the Society to some of the unusual effects of this remedy.

It is well known that the continued use of the sulphate produced congestion of the conjunctiva, and even vascularity of the cornea in exceptional cases. It is not, perhaps, so well known by general practitioners, that it produces, in some patients, a very distressing eczematous irritation of the lids. It is, probably, not generally known that, dropped into an eye with glaucomatous tendencies, or one in which there is a choroidal tumor, it is liable to produce, suddenly, very great pain, with intraocular inflammation.

In the use of so violent a poison even locally, great care should be observed, lest too much of the liquid pass through the nasal ducts. As often as the drop is instilled into the eye, the lids should be at once carefully wiped, so that the fluid which does not adhere to the mucous membrane may be removed. Generally, the quarter of an ordinary drop, applied to the internal angle of the eye, by means of a large probe, is sufficient. It should be borne in mind that some persons are very susceptible to the influence of atropine. A slight fever, with a flush of the cheeks, will occasionally be produced by this remedy, even in minute quantities.

In two cases in which I had extracted cataract, the atropine had been used, as far as was known, precisely as in other cases. On the third day, in each case, violent mania occurred. A writer on cataract reports two cases of mania after extraction. I heard, recently, of two other such cases in the practice of a skilled operator. It had occurred to me that, possibly, all these patients were either very susceptible to the effects of atrophia, or, in some way, received larger quantities into the system than was intended.

### CALOMEL.

A fact, not generally mentioned in our works on diseases of the eye, and not very generally known in the profession, I believe, is worthy of notice in reference to the local use of calomel. This remedy is of very great benefit in certain diseases, especially of the cornea, and much used. It should be remembered that it can be placed on the conjunctiva and cornea, not without danger, when the patient is taking the iodide of potash internally. The salts in the tears, and the calomel, often form a caustic sufficiently strong to produce quite a deep and painful eschar.

### HANCOCK'S OPERATION.

This operation was devised as a substitute for iridectomy in the treatment of glaucoma. The author described the good effects of the operation to division of the ciliary muscle. A late member of this Society had very often performed the operation in a variety of diseases, and presented a paper on the subject at one of its meetings. He ascribed the benefits of the operation, as did Hancock, to the division of the ciliary ring. In conversation with the author of the paper alluded to, I was induced to perform the operation in painful abscess and ulcers of the cornea, believing, however, that the incision was simply a modified paracentesis, and that the " division of the ciliary muscle " did not take place ; since the direction of the cut was parallel to, but not across, the principal fibres of this muscle.

The evacuation of the aqueous humor is of great value in several conditions of the eye. As usually performed, through a minute puncture in the cornea, the wound heals at once, allowing the aqueous humor to accumulate in a very short time. Hancock's incision is three or four lines long, through the sclerotic perpendicular to the circumference of the cornea, but extending fairly into the anterior chamber. In this way, the aqueous, and some of the vitreous humor, is discharged—the wound, however, remains open some hours, allowing the fluid to drain away as fast as it accumulates. I have many times derived very great benefit from this little operation.

In introducing the triangular cataract-knife—perhaps the most convenient instrument in making the incision—care should be taken that the point be carried backward and outward from the axis of the globe, to avoid injuring the edge of the crystalline lens.

SYMPATHETIC OPHTHALMITIS.

I trust the Society will pardon me for again alluding to this subject. Its importance is impressed upon my mind from the fact that I have recently seen several cases of total blindness, in which vain attempts, by practitioners, to relieve the painful symptoms in an injured and blind eye resulted in the total loss of the other eye.

Almost any injury or disease of the globe—especially the presence of a foreign body within it—which leaves the globe, for a period, inflamed, possibly atrophied, with marked tenderness on pressure over the ciliary region, is liable to develop intraocular inflammation in the other eye—an inflammation which can seldom be arrested when once aroused. Since the danger is so terrible, there should be no delay in removing the diseased eye at an early period, while the other eye is yet sound.

ANEURISM OF THE ORBIT.

M. F., æt. twenty-two, received, last January, in a drunken quarrel, a blow from an iron weight, near the external angle of the right eye. Both lids were severely lacerated and extensively torn from the underlying tissues. The globe was also ruptured. The patient at once came under the care of a prudent practitioner,

In consultation with the physician, at the patient's residence, some days after, I found the lids and conjunctiva enormously swollen, the wound of the lids having been carefully adjusted with sutures. Simple, cooling lotions were applied for a week. Pain had been very slight since the injury. I was again called to the patient at the end of a week, and found the swelling as extensive as before, with considerable tenderness. As the large wound in the cornea remained open, with the iris protruding, I advised the removal of the whole cornea and iris. At the patient's request, he was taken to the Eye and Ear Infirmary.

I must here state that it was only when the patient reached the Infirmary that I discovered an extensive pulsating movement in the orbit and a remarkable bruit, synchronous with the pulse, which the patient stated he had observed a couple of days after the injury, although he had said nothing regarding it. I removed the cornea and iris, applied gentle pressure, and gave the patient tinct. verat. viride and tinct. ergotæ, in large doses, since a traumatic aneurism of the orbit, under my care, some years since, recovered under the use of these remedies.

At the end of a month the swelling was greatly reduced, the pulsation and bruit very much less distinct, in fact, scarcely perceptive when the patient stood erect. For a few days the patient was confident a cure was effected. The symptoms, however, soon returned without apparent cause. In addition, an artery along the brow to the temples became much enlarged, pulsating with violence, although the bruit was much less distinct than before.

In consultation with Prof. Powell, it was decided to reduce the patient's diet to a minimum in quantity and quality. After a trial of two weeks, the patient taking daily but a small quantity of bread and water, the symptoms were, if possible, more marked than before. It was finally decided to inject a half drachm of sol. persulph ferri, which was done by Prof. Powell, by means of a subcutaneous injection syringe, without the administration of an anæsthetic. The point of the syringe was carried deep into the orbit, and, probably, fairly into the aneurismal sack, since all the symptoms subsided at once. For some days there were considerable swelling and pain, extending over the cheek, down to the jaw and neck. At the end of a fortnight all symptoms had disappeared, when the patient was discharged well.

